# Recurrence dynamics after curative surgery in patients with invasive mucinous adenocarcinoma of the lung

**DOI:** 10.1186/s13244-022-01208-5

**Published:** 2022-04-05

**Authors:** Hyun Jung Yoon, Jun Kang, Ho Yun Lee, Min A. Lee, Na Young Hwang, Hong Kwan Kim, Jhingook Kim

**Affiliations:** 1 Department of Radiology and Center for Imaging Science, Samsung Medical Center, Sungkyunkwan University School of Medicine, 81 Irwon-Ro, Gangnam-Gu, Seoul, 06351 South Korea; 2 Department of Radiology, Veterans Health Service Medical Center, Seoul, South Korea; 3 Department of Hospital Pathology, Seoul St. Mary’s Hospital, College of Medicine, The Catholic University of Korea, Seoul, South Korea; 4 Department of Health Sciences and Technology, SAIHST, Sungkyunkwan University, Seoul, 06351 South Korea; 5 Samsung Cancer Research Institute, Samsung Medical Center, Seoul, South Korea; 6 Department of Thoracic Surgery, Samsung Medical Center, Sungkyunkwan University School of Medicine, Seoul, South Korea

**Keywords:** Mucinous adenocarcinoma, Recurrence, Hazard rate, CT, STAS

## Abstract

**Background:**

We investigated the patterns and timing of recurrence and death as well as prognostic factors based on clinicopathological and radiological factors in patients who underwent surgical treatment for invasive mucinous adenocarcinoma (IMA).

**Methods:**

We reviewed clinicopathological findings including spread through air spaces (STAS) and CT findings of IMA such as morphology, solidity, margin, well-defined heterogeneous ground-glass opacity, CT angiogram, and air bronchogram signs from 121 consecutive patients who underwent surgical resection. Prognostic factors for disease-free survival (DFS) and overall survival (OS) were identified. Hazard rate analyses were performed for the survival dynamics.

**Results:**

T stage (hazard ratio [HR] = 4.102, *p* = 0.03), N stage (N2 vs. N0, HR = 7.653, *p* < 0.001), and consolidative CT morphology (HR = 3.556, *p* = 0.008) remained independent predictors for DFS. Age (HR = 1.110, *p* = 0.002), smoking (HR = 12.893, *p* < 0.001), T stage (HR = 13.005, *p* = 0.006), N stage (N2 vs. N0, HR = 7.653, *p* = 0.004), STAS (HR = 7.463, *p* = 0.008), and consolidative CT morphology (HR = 6.779, *p* = 0.007) remained independent predictors for OS. Consolidative morphology, higher T and N stage, and presence of STAS revealed initial sharp peaks after steep decline of the hazard rate curves for recurrence or death in follow-up period.

**Conclusions:**

Consolidative morphology, higher T and N stage, smoking, and STAS were indicators of significantly greater risk of early recurrence or death in patients with IMA. Thus, these findings could be incorporated into future surveillance strategies.

**Supplementary Information:**

The online version contains supplementary material available at 10.1186/s13244-022-01208-5.

## Key points

- Lung IMA patients with consolidative CT morphology, higher T stage, and higher N stage were significantly more prone to recurrence and the dynamics of recurrence after surgical treatment showed higher hazard rates and peaks that appeared during the early phase of follow-up. Our findings provide information relevant to the selection of patients at higher risk of recurrence and death as well as the timing of surveillance studies.

## Background

Invasive mucinous adenocarcinoma (IMA) is a variant of invasive adenocarcinoma of the lung that accounts for approximately 5%–10% of lung adenocarcinomas [1, 2]. IMA is characterized by invasive columnar or goblet cell patterns with basally located nuclei and abundant intracytoplasmic mucin [3]. IMA has remarkably distinct molecular, clinicopathological, and radiologic characteristics compared with other subtypes of adenocarcinoma [4–6].

In terms of prognosis, survival data for patients with IMA are limited due to its low incidence. Also, limited studies with conflicting results have revealed that the prognosis of IMA is not as well typified as that of nonmucinous adenocarcinoma [2]. Moreover, pneumonic type IMA is characterized by multifocal and multilobar involvement [7], and subsequently, recurrence during follow-up after surgery is often delayed because of the intrinsic difficulty in distinguishing infectious pneumonia or drug-induced pneumonitis manifesting as a cyptogenic organizing pneumonia pattern and recurrence of IMA on the basis of postoperative follow-up CT findings [8–11]. In conjunction with these findings, a few studies have suggested that radiologic features such as consolidative morphology as well as tumor margin and tumor density on computed tomography (CT) are significant prognostic indicators [8–14]. Thus, understanding recurrence patterns and incidence information for pneumonic type IMA is becoming more important for predicting survival.

Spread through air spaces (STAS), which has recently been recognized as a pattern of invasion in lung cancer, is a potential biomarker for worse prognosis in IMA patients [9–11, 15–17]. Several previous studies demonstrated that IMA showed a higher incidence of STAS (50–72.3%) [12, 18, 19] compared with patients with nonmucinous adenocarcinoma (approximately 14.8–47.6%) [15, 16]. The higher incidence of STAS suggests the presence of intraalveolar tumor cells with detached primary focus and the possibility of pathogenic associations with intrapulmonary aerogenous metastasis of IMA.

However, due to the extremely wide spectrum of tumor behavior in IMA the survival outcomes of IMA patients are not adequately reflected by recognized prognostic factors in clinical practice. Moreover, these indicators do not provide direct information regarding changes in event probabilities over time (i.e., event dynamics), which can be estimated by calculating event-specific hazard rates over the follow-up period [20]. Thus, in the present study, we investigated the patterns and timing of recurrence and death as well as prognostic factors based on clinical, radiological and pathological factors in patients who underwent surgical treatment for IMA.

## Methods

This retrospective study was approved by our institutional review board (approval 2015-03-089), and the requirement for informed consent was waived.

### Study population

All patients who had undergone curative surgery for lung IMA in the surgical database of the Department of Thoracic Surgery within a tertiary referral cancer center at our institution were included in the study. We identified 138 patients between February 1998 and November 2012. For prognostic evaluation of IMA patients, 17 patients were excluded for the following reasons: (1) synchronous or metachronous lung cancer (*n* = 6); (2) concomitant presence of malignancy other than lung cancer (*n* = 5); and (3) insufficient quality of CT imaging for accurate review. Finally, a total of 121 patients with completely resected solitary IMAs were included in this study.

Data for age, sex, smoking history, type of surgery, and neoadjuvant or adjuvant therapy were collected using electronic medical records of clinical factors. Details of the protocol for surgical therapy were described in our previous study [12]. In terms of pathologic presentation, surgical margin distance of tumor, T classification, lymph node (LN) metastasis, pathologic stage, and histologic differentiation of tumor were evaluated. The TNM classification of the American Joint Committee on Cancer Staging Manual (seventh edition) was used for pathological staging [21]. Epidermal growth factor receptor (EGFR) mutations status was also recorded if available.

### Radiologic analysis

**Fig. 1:**
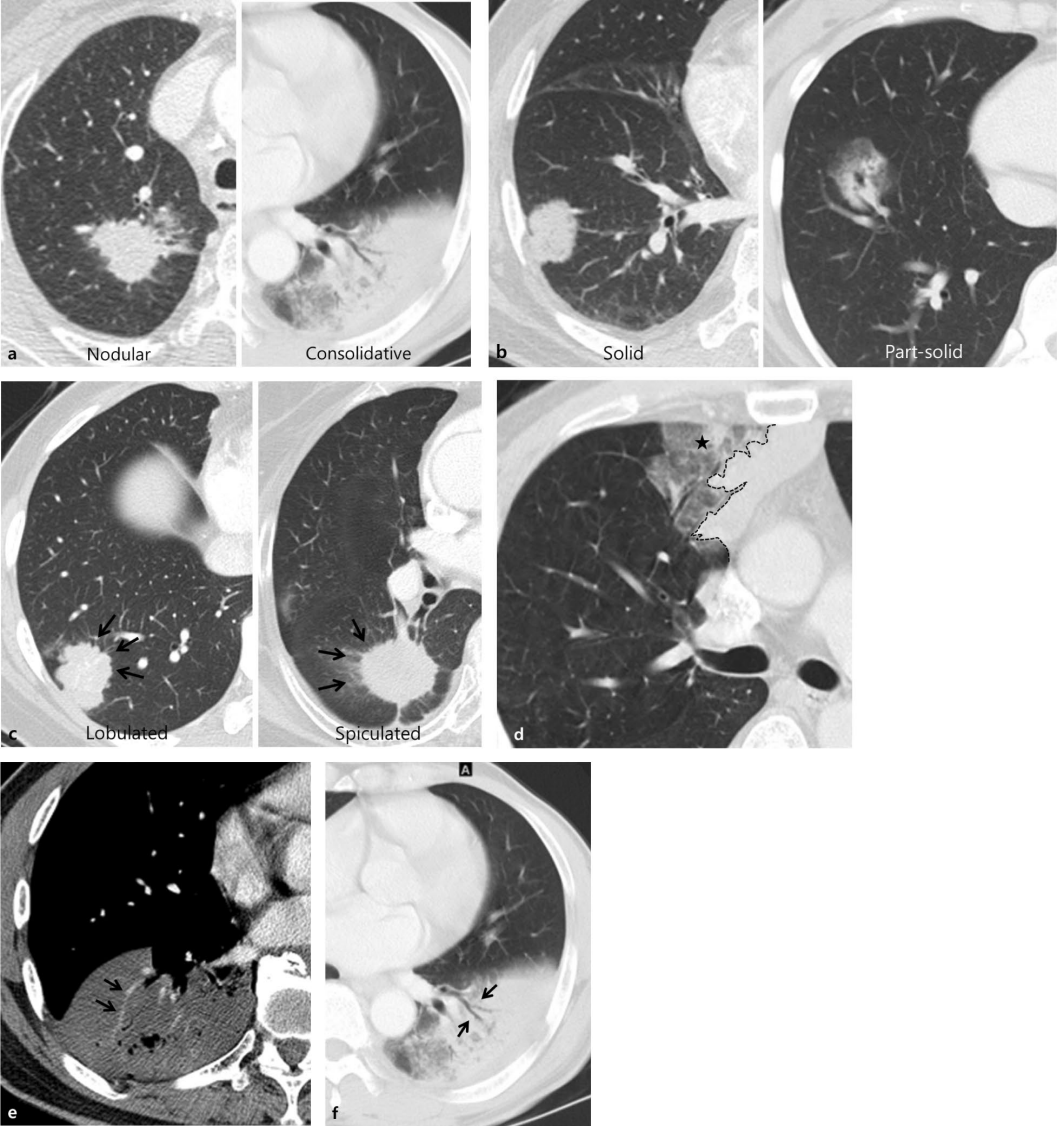
Examples of CT images of morphology (**a**), solidity (**b**), margin characteristics (**c**), well-defined heterogeneous ground-glass opacity (**d**), CT angiogram sign (**e**), CT air-bronchogram sign (**f**) showing typical features of invasive mucinous adenocarcinomas of the lung. Well-defined heterogeneous ground-glass opacity (asterisk) defined as the combination of consolidation and ground-glass opacity detected beyond the imaginary margin of the lesion (dashed line)

Two board-certified thoracic radiologists independently reviewed preoperative CT images. Discrepancies after the first independent review were resolved through the second review, and decisions were reached by consensus. CT scans with intravenous contrast enhancement were considered of diagnostic quality if they were acquired or reconstructed to 2.5 mm thickness or less without artifacts [22]. CT factors evaluated for all IMAs were morphology (nodular vs. consolidative), solidity (solid vs. part-solid), margin characteristics including lobulated and spiculated margins, presence of well-defined heterogeneous ground-glass opacity (GGO), CT angiogram, and air bronchogram signs. Representative images are shown in Fig. 1.

### Histopathologic analysis

An experienced lung pathologist previously reviewed all slides using the Aperio Slide Scanning System (ScanScope T3; Aperio Technologies Inc., Vista, CA, USA), producing high-resolution digital images (0.25 lm/pixel at 40) [12]. Histopathologic analyses included presence of STAS, aerogenous spread, and mucin. STAS was defined as detachment of small solid cell nests (at least five tumor cells) within the airspace in the lung parenchyma beyond the edge of the main tumor [15] (Additional file 1:: S1 Appendix). Aerogenous spread was defined as discontinuous spread of lepidic pattern more than 3 mm from the main mass, discrete micro-nodules of lepidic pattern apart from the main mass, and intermingling with lepidic pattern of tumor and non-tumor alveolar structure within the main tumor.

### Follow-up scheme and recurrence pattern analysis

Patients were strictly followed up every three months for the first two years after surgery and every six months thereafter with an annual CT scan. They were also regularly evaluated via an interval history assessment, physical examination, blood tests, and chest radiography at each visit. In 1998, bone scanning was integrated into the routine annual surveillance program, but it soon replaced in 2002 by PET-CT when it was introduced at our institution. Whole-brain CT or brain magnetic resonance imaging and other imaging techniques were performed as indicated by the patients’ symptoms. In cases requiring a pathological diagnosis to support the clinical diagnosis and the decision to initiate treatment, we performed invasive procedures including CT-guided needle biopsy, mediastinoscopy, and bronchoscopy. For patients lost to follow-up, a telephone interview was conducted to determine late outcomes.

Recurrence was confirmed with a combination of clinical, radiologic, pathologic, and surgical findings. The postoperative recurrence pattern was divided into two patterns: loco-regional and distant recurrence. Loco-regional recurrence was defined as evidence of a tumor at the surgical margin of the original tumor in the same lobe or a second ipsilateral lobe, in an ipsilateral hilar LN, or ipsilateral mediastinal LN. Distant recurrence was defined as evidence of a tumor in the contralateral lung, contralateral mediastinal LN, ipsilateral supraclavicular LN, or outside the hemithorax [12, 23]. All sites of disease recurrence were recorded.

Additionally, we divided patients who had postoperative recurrence into two subtypes: oligometastases and polymetastases. Oligometastases have better prognosis than polymetastases in several solid tumors, including lung cancer; thus, subtype is correlated with patient management [24, 25]. Oligometastases are defined as 1–5 distant metastases that can be treated by local therapy to achieve long-term survival or even cure [26]. The recurrence subtype was included as a clinical factor for prognostic analysis.

### Statistical analysis

Disease-free survival (DFS) and overall survival (OS) were determined using the electronic medical records of our institution. DFS was calculated from the date of surgery until either the date of recurrence (event; defined as local tumor recurrence, distant metastasis, or death), or until the date that the patient was last known to be free of recurrence (censored). OS was calculated from the date of surgery until either death from any cause (event) or the date the patient was last known to be alive (censored).

Cox proportional hazards regression models were used to assess the prognostic value of all clinical (sex, age, smoking, surgical procedure, adjuvant treatment, and recurrence subtype), pathological (surgical bronchial margin, T and N stage, tumor differentiation, aerogenous spread, mucin, STAS, and *EGFR* mutation status), and CT (CT morphology, solidity, well defined heterogenous GGO, lobulated or spiculated margin, CT air-bronchogram, and CT angiogram sign) factors in IMA patients on univariate analysis. *p* values < 0.1 were considered statistically significant on univariate analysis, and adjusted multivariate forward-conditional Cox regression analysis was used to identify independent risk factors. *p* values < 0.05 were considered statistically significant on multivariate analysis. Receiver operating characteristic (ROC) analysis with area under the curve (AUC) calculation was conducted to compare sensitivity and specificity for prediction of DFS and OS by our proposed risk score model with fivefold validation. Calibration plots that describe the level of agreement between predicted and observed survival were used to assess the performance of the survival prediction model. Kaplan–Meier analysis with a log-rank test was used to compare the DFS and OS according to significant risk factors. Hazard rate curves were drawn to measure the impact of the selected clinical, radiological, and pathological variables on disease recurrence and death during follow-up after surgery. All data analyses were performed using R (R version 3.5.1, Vienna, Austria).

For CT morphology (nodular vs. consolidative), we randomly extracted 30 cases and calculated the interobserver agreement using the kappa statistic to evaluate the agreement between the two readers. A kappa statistic of 0.81–1.00 indicates an excellent agreement; 0.61–0.80, substantial agreement; 0.41–0.60, moderate agreement; 0.21–0.40, fair agreement; and 0.00–0.20, poor agreement [27].

## Results

### Demographics and CT findings

The distributions of clinicopathological and radiologic factors according to survival events of all solitary IMA patients are shown in Table 1. A total of 113 patients (93.4%) underwent lobectomy, and eight patients (6.6%) underwent sublobar resection (limited resection). The median tumor size was 3.15 cm, ranging from 0.5 to 12.6 cm in diameter. The median tumor size of the 91 nodular type tumors was 2.6 cm (IQR [interquartile range], 1.7–3.9 cm), and the median tumor size of the 30 consolidative type tumors was 5.6 cm (IQR 3.9–8.5 cm). The median surgical bronchial margin, determined as the shortest centimeter distance between the tumor margin and resected bronchus, was 1.4 cm. The median number of LNs dissected during surgery was 15, and 15 patients (12.4%) had nodal metastasis. Among 121 patients, 29 patients (24%) received adjuvant or neoadjuvant therapy and 92 patients (76%) did not. The types of neoadjuvant or adjuvant therapy were adjuvant chemotherapy in 20 patients (69%), adjuvant radiotherapy in four patients (13.8%), adjuvant CCRT (concurrent chemoradiotherapy) in one patient (3.4%), neoadjuvant chemotherapy plus adjuvant chemotherapy in three patients (10.3%), and neoadjuvant CCRT plus adjuvant radiotherapy in one patient (3.4%). The interobserver agreement between the two readers for CT morphology was substantial (kappa value of 0.78).

**Table 1:** Patient distribution according to clinicopathological and radiologic factors

No. of patients	Total = 121
**Clinical factor**	
Sex (%)	
Male	56 (46.3)
Female	65 (53.7)
Age, median (IQR)	58 (52–68)
Smoking (%)	
Never	82 (67.8)
Ever	39 (32.2)
Surgical procedure (%)	
Sublobar resection	8 (6.6)
Lobectomy	113 (93.4)
Neoadjuvant or adjuvant treatment (%)	
No	92 (76)
Yes	29 (24.0)
**CT factor**	
CT morphology (%)	
Nodular type	91 (75.2)
Consolidative type	30 (24.8)
Solidity (%)	
Solid	63 (52.1)
Part-solid	58 (47.9)
Well-defined heterogenous GGO (%)	15 (12.4)
Lobulated margin (%)	47 (38.8)
Spiculated margin (%)	38 (31.4)
CT air-bronchogram (%)	70 (57.9)
CT angiogram sign (%)	25 (20.7)
**Pathologic factor**	
Surgical bronchial margin (%)	
> 1 cm	98 (81)
≤ 1 cm	9 (7.4)
NA	14 (11.6)
T stage (%)	1.6 (0.5)
1A/B	53 (43.8)
2A/B	67 (55.4)
3	1 (0.8)
N stage (%)	
0	106 (87.6)
1	5 (4.1)
2	10 (8.3)
No. of LNs dissected, median (IQR)	15 (9–21)
TNM stage (%)	
I	82 (67.8)
II	24 (19.8)
III	15 (12.4)
Tumor differentiation (%)	
Well	89 (73.6)
Moderate	25 (20.7)
Poor	7 (5.8)
Aerogenous spread (%)	
No	52 (43.0)
Yes	62 (51.2)
NA	7 (5.8)
Mucin (%)	
No	39 (32.2)
Yes	71 (58.7)
NA	11 (9.1)
STAS (%)	
No	31 (25.6)
Yes	79 (65.3)
NA	11 (9.1)
*EGFR* mutation (%)	
No	11 (9.1)
Yes	21 (17.3)
NA	89 (73.6)
**Prognosis**	
OS event, yes (median follow-up period 81.4 [IQR, 41.8–109.5 months])	31 (25.6)
DFS event, yes (median follow-up period 59.8 [IQR, 24.6–104.6 months])	35 (28.9)

*IQR* interquartile range, *CT* computed tomography, *GGO* ground glass opacity, *LN* lymph node, *NA* not available, *STAS* spread through air spaces, *EGFR* epidermal growth factor receptor, *OS* overall survival, *DFS* disease-free survival

The median follow-up period without recurrence was 59.8 months (IQR 24.6–104.6 months), and total median follow-up period was 81.4 months (IQR 41.8–109.5 months). The median number of times for out-patient department visits was 15 times (IQR 10–24 times). The 5-year DFS and OS rates were 68.5% and 77%, respectively. The median DFS and OS of all patients were 66.2 and 81.8 months, respectively. The median DFS and OS of 29 patients who received adjuvant or neoadjuvant therapy were 70.5 and 81.4, months, respectively. The median DFS and OS of 92 patients who received only surgical treatment were 59.7 and 81.8 months, respectively.

A total of 35 patients (28.9%) experienced recurrence during the follow-up period. During the follow-up period, a total of 31 patients (25.6%) died. Among them, 25 patients died as a result of metastasis from lung mucinous adenocarcinoma, four patients died of old age, one patient died of progression of interstitial lung disease, and one patient died of pneumonia.

The eight patients who underwent sublobar resection did not show disease recurrence nor death during follow-up period. Detailed follow-up information with respective demographic and tumor characteristics is shown in Table S1.

### Recurrence patterns

Among the 35 patients with recurrence, four had recurrence in a different ipsilateral lobe, indicating locoregional metastasis, and 31 patients had distant metastasis. Among the 31 patients with distant metastasis, 23 patients had contralateral lung and/or pleural metastasis, four had brain metastasis, two had bone and brain metastasis, one had brain and mediastinal LN metastasis, and one had bone and mediastinal LN metastasis. When the 35 patients with recurrence were divided into two subtypes, oligometastases and polymetastases, 11 patients had oligometastases and 24 patients had polymetastases. Among the 11 patients with oligometastases, three had extrathoracic metastasis, and among the 24 patients with polymetastases, five had extrathoracic metastasis. The patterns of recurrence and recurrence sites are shown in Table 2.

**Table 2:** Patterns of recurrence and recurrence sites

	No. of patients (%)
Loco-regional recurrence	4 (11.4)
Distant recurrence	31 (88.6)
Site of disease recurrence	
Lung	23 (65.7)
Pleura	4 (11.4)
Brain	7 (20)
Bone	3 (8.6)
Mediastinal LNs	2 (5.7)
Number of recurrence or metastasis	
Oligo	11 (31.4)
Poly	24 (68.6)

*LN* lymph node

### Prognostic analysis

The results of univariate and multivariate analyses of DFS and OS in solitary IMA patients are shown in Tables 3 and 4. For DFS, univariate analyses found that older age (*p* = 0.017), history of adjuvant treatment (*p* = 0.079), shorter surgical bronchial margin (*p* = 0.027), higher T stage (*p* < 0.001), higher N stage (N1 vs. N0, *p* = 0.028 and N2 vs. N0, *p* < 0.001, respectively), presence of mucin (*p* = 0.056), consolidative CT morphology (*p* < 0.001), and well-defined heterogenous GGO on CT (*p* = 0.018) were associated with worse prognosis. In adjusted multivariate analysis, higher T stage (hazard ratio [HR] = 4.102, *p* = 0.03), higher N stage (N1 vs. N0, HR = 4.131, *p* = 0.045, N2 vs. N0, HR = 7.653, *p* < 0.001, respectively), and consolidative CT morphology (HR = 3.556, *p* = 0.008) remained independent predictors for DFS (Table 3). The predictive model for prediction of DFS showed performance with AUC of 0.887 at 36 months and 0.807 at 60 months (Additional file 1: Fig. S1A). ROC analysis of sensitivity and specificity for prediction of DFS with fivefold validation at several time points is shown in Fig. S2A. The AUC was highest at 0.883 at 25, 26, and 27 months.

**Table 3:** Results of univariate and multivariate analyses of disease-free survival

Variable	Univariate				Multivariate			
	HR	95% CI		*p* value	HR	95% CI		*p* value
Clinical factor								
Sex, female	1.088	0.557	2.125	0.806				
Age (years), older	1.039	1.007	1.072	**0.017**	1.026	0.986	1.067	0.204
Smoking, present	1.221	0.607	2.456	0.575				
Surgical procedure, lobectomy	–	0.000	Inf	0.997				
Adjuvant treatment, present	1.870	0.930	3.760	**0.079**	1.293	0.554	3.017	0.552
CT factor								
CT morphology, consolidative type	3.404	1.747	6.632	** < 0.001**	3.556	1.395	9.063	**0.008**
Solidity, part-solid type	1.163	0.599	2.257	0.655				
Well-defined heterogenous GGO	2.604	1.181	5.743	**0.018**	1.058	0.362	3.097	0.918
Lobulated margin	1.472	0.758	2.856	0.253				
Spiculated margin	1.114	0.554	2.239	0.763				
CT air bronchogram	0.964	0.494	1.884	0.916				
CT angiogram sign, present	0.861	0.334	2.220	0.757				
Pathologic factor								
Surgical bronchial margin, ≤ 1 cm	2.956	1.132	7.720	**0.027**	1.145	0.358	3.662	0.819
T stage, higher	6.329	2.451	16.343	** < 0.001**	4.102	1.151	14.627	**0.030**
N stage, higher, N2 vs. N0^*^	7.332	3.241	16.586	** < 0.001**	7.295	2.523	21.092	** < 0.001**
Tumor differentiation, poor, poor vs. well^†^	2.768	0.823	9.306	0.100				
Aerogenous spread, present	1.523	0.749	3.096	0.245				
Mucin, present	0.513	0.259	1.016	**0.056**	0.616	0.257	1.475	0.276
STAS, present	1.469	0.635	3.399	0.368				
* EGFR* mutation, yes	1.116	0.458	2.716	0.809				

Bold numbers indicate statistical significance (*p* < 0.10 on univariate analysis and *p* < 0.05 on multivariate analysis). *HR* hazard ratio, *CI* confidence interval, *CT* computed tomography, *STAS* spread through air spaces, *EGFR* epidermal growth factor receptor

^*^’N0’ was regarded as reference

^†^’Well differentiated’ was regarded as reference

**Table 4:** Results of univariate and multivariate analyses of overall survival

Variable	Univariate				Multivariate			
	HR	95% CI		*p* value	HR	95% CI		*p* value
Clinical factor								
Sex, female	0.647	0.319	1.314	0.228				
Age (years), older	1.086	1.045	1.127	** < 0.001**	1.110	1.040	1.185	**0.002**
Smoking, present	1.977	0.974	4.012	**0.059**	12.893	3.226	51.532	** < 0.001**
Surgical procedure, lobectomy	–	0.000	Inf	0.997				
Adjuvant treatment, present	1.410	0.649	3.064	0.385				
Recurrence subtype, poly^*^	6.872	1.996	23.660	**0.002**				
CT factor								
CT morphology, consolidative type	3.181	1.571	6.442	**0.001**	6.779	1.675	27.444	**0.007**
Solidity, part-solid type	1.196	0.591	2.420	0.618				
Well-defined heterogenous GGO	2.341	1.006	5.444	**0.048**	1.793	0.473	6.797	0.391
Lobulated margin	1.068	0.523	2.180	0.857				
Spiculated margin	0.716	0.320	1.602	0.417				
CT air-bronchogram	1.010	0.495	2.062	0.978				
CT angiogram sign, present	1.049	0.403	2.732	0.922				
Pathologic factor								
Surgical bronchial margin, ≤ 1 cm	3.147	1.185	8.358	**0.021**	2.699	0.431	16.901	0.289
T stage, higher, T2/3 vs. T1	6.242	2.183	17.850	**0.001**	13.005	2.057	82.213	**0.006**
N stage, higher, N2 vs. N0^†^	7.097	2.959	17.020	** < 0.001**	7.653	1.927	30.386	**0.004**
Tumor differentiation, poor, poor vs. well^‡^	5.414	2.006	14.612	**0.001**	5.071	0.697	36.924	0.109
Aerogenous spread, present	1.018	0.490	2.116	0.962				
Mucin, present	0.458	0.218	0.963	**0.039**	1.576	0.460	5.404	0.469
STAS, present	2.501	0.864	7.239	**0.091**	7.463	1.702	32.729	**0.008**
* EGFR* mutation, yes	0.944	0.326	2.730	0.915				

Bold numbers indicate statistical significance (*p* < 0.10 on univariate analysis and *p* < 0.05 on multivariate analysis). *HR* hazard ratio, *CI* confidence interval, *CT* computed tomography, *STAS* spread through air spaces, *EGFR* epidermal growth factor receptor

^*^Multivariate analysis could not be performed due to missing data; †’N0’ was regarded as reference; ^‡^’Well differentiation’ was regarded as reference

For OS, univariate analyses with the Cox proportional hazard model found that older age (*p* < 0.001), history of smoking (*p* = 0.059), polymetastases type tumor recurrence (*p* = 0.002), shorter surgical bronchial margin (*p* = 0.021), higher T stage (*p* = 0.001), higher N stage (N1 vs. N0, *p* = 0.068 and N2 vs. N0, *p* < 0.001, respectively), poorly differentiated tumor (*p* = 0.001), presence of mucin (*p* = 0.039), presence of STAS (*p* = 0.091), consolidative CT morphology (*p* = 0.001), and well defined heterogenous GGO on CT (*p* = 0.048) were associated with worse prognosis. In adjusted multivariate analysis to identify independent prognostic factors excluding recurrence subtype due to missing data, older age (HR = 1.110, *p* = 0.002), history of smoking (HR = 12.893, *p* < 0.001), higher T stage (HR = 13.005, *p* = 0.006), higher N stage (N2 vs. N0, HR = 7.653, *p* = 0.004), presence of STAS (HR = 7.463, *p* = 0.008), and consolidative CT morphology (HR = 6.779, *p* = 0.007) remained independent predictors for OS (Table 4). The predictive model for prediction of OS showed AUC of 0.9 at 36 months and 0.896 at 60 months (Additional file 1: Fig. S1B). The ROC analysis of the sensitivity and specificity for prediction of OS with five-fold validation at several time points is shown in Fig. S2B. The AUC was highest at 0.89 at 31 and 32 months. The calibration plot of prediction model for OS and DFS at 36 and 60 months showed good agreement between predicted and observed survival (Fig. S3).

Kaplan–Meier analysis with a log-rank test demonstrated a lower DFS rates in patients with consolidative CT morphology (*p* < 0.001), higher T stage (*p* < 0.001, T1 vs.T2), and higher N stage (*p* = 0.004, N0 vs.N1 and *p* < 0.001, N0 vs. N2) and there was statistical significance. Lower OS rates were also noted in patients with histories of smoking (*p* = 0.054), STAS (*p* = 0.08), consolidative CT morphology (*p* = 0.001), higher T stage (*p* < 0.001, T1 vs.T2), higher N stage (*p* = 0.004, N0 vs.N1 and *p* < 0.001, N0 vs. N2), and polymetastases (*p* = 0.006), although there was no statistically significant difference in smoking and STAS (Additional file 1: Fig. S4 and S5).

### Hazard rate analysis

**Fig. 2:**
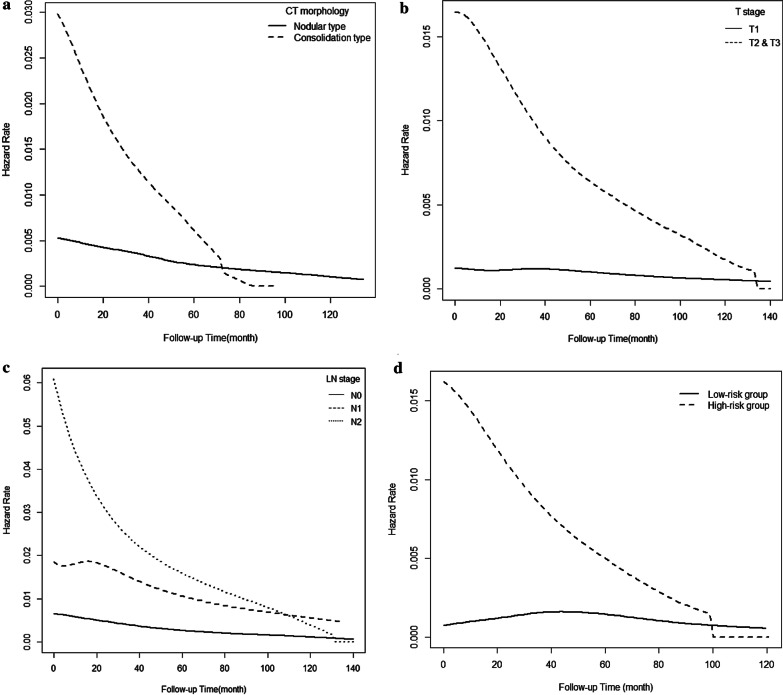
Comparison of recurrence hazard rate according to CT morphology (**a**), T stage (**b**), N stage (**c**), and recurrence hazard rate comparing groups with at least one of the significantly unfavorable factors (consolidative morphology, higher T and N stage [high risk group]) and those without (low risk group) (**d**) in patients with invasive mucinous adenocarcinoma

**Fig. 3:**
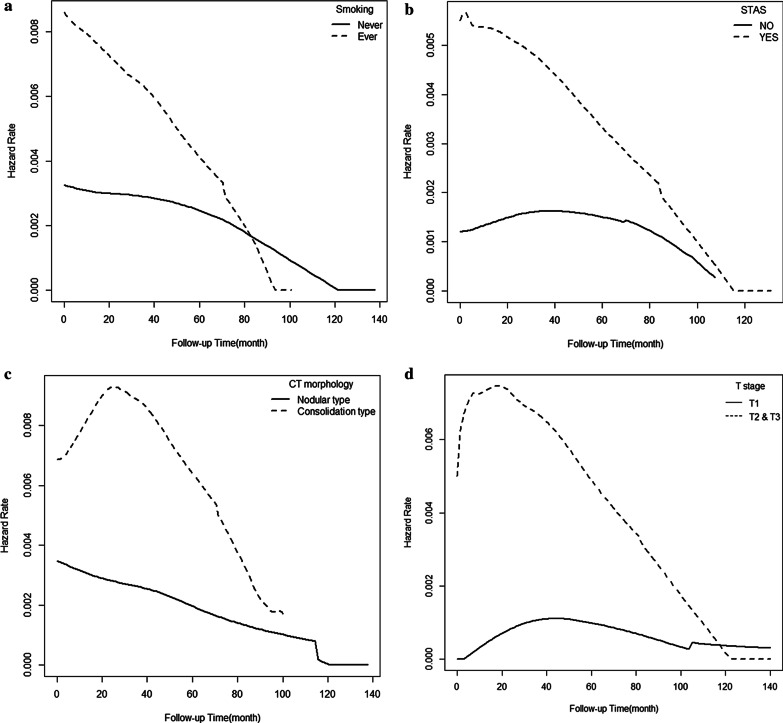

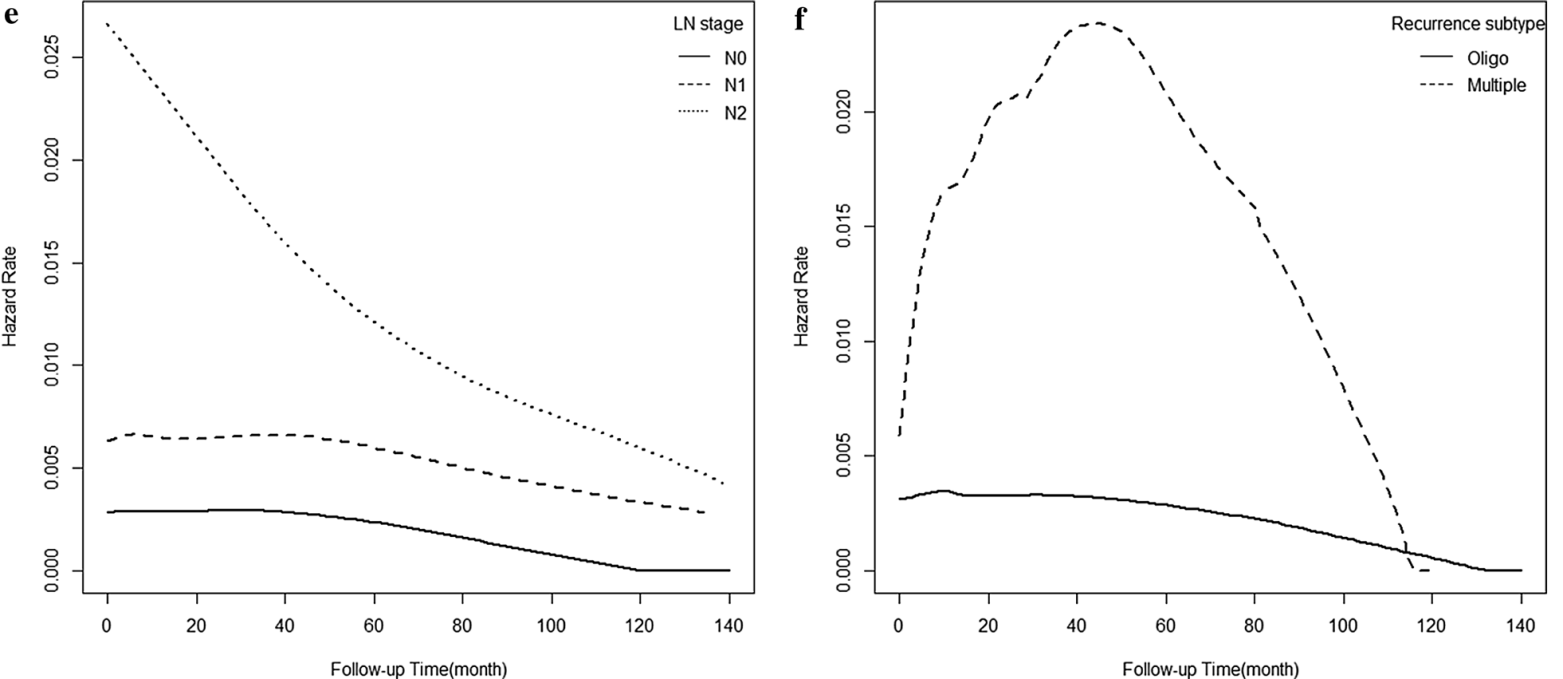
Comparison of the death hazard rate according to smoking (**a**), STAS (**b**), consolidative CT morphology (**c**), higher T stage (**d**), higher N stage (**e**), and recurrence subtype (**f**) in patients with invasive mucinous adenocarcinoma

Hazard rate curves for recurrence and death according to independently significant prognostic factors are shown in Figs. 2 and 3. For DFS, patients with consolidative CT morphology, higher T stage, and higher N stage had higher hazard rates, and the peaks of the hazard rate curves appeared in the initial phase during the follow-up period and steeply declined rapidly over the first 40 months. We additionally performed the hazard rate analysis for DFS comparing IMAs with at least one of the significantly unfavorable factors (consolidative morphology, higher T and N stage [high risk group]) and those without (low risk group). The hazard rate curves for recurrence were similar to those of each significant unfavorable factor (Fig. 2D). Although STAS was not significant prognostic factor (*p* = 0.368 in univariate analysis) for DFS, the peaks of the recurrence hazard rate curve showed higher in presence of STAS than in absence of STAS (Additional file 1: Fig. S6). For OS, comparisons of death hazard rates revealed that patients with histories of smoking, STAS, consolidative CT morphology, and higher T and N stages had higher hazard rates, and the peaks of the hazard rate curves appeared in the early phase during the follow-up period and declined rapidly until reaching a nadir. In contrast, recurrence or death hazard rate curves for never smoker, absence of STAS, nodular CT morphology, and lower T and N stage were relatively flattened. The curve for recurrence subtype illustrated that polymetastases had higher hazard rates than oligometastases and death peaked at 40 months after surgery. We could not compare the hazard rate curve for death between high risk and low risk group since all 13 low risk group for OS are still alive.

## Discussion

In our study, higher T stage, higher N stage, and consolidative CT morphology were independent predictors of DFS, and the model for prediction of DFS showed good performance with AUC of 0.887 at 36 months and 0.807 at 60 months. Age, history of smoking, higher T stage, higher N stage, presence of STAS, and consolidative CT morphology were independent predictors of OS. The predictive model of OS showed good performance with AUC of 0.9 at 36 months and 0.896 at 60 months. These results are in line with those of previous studies [9, 12, 13]. In addition to efforts to identify prognostic factors for developing recurrence or death in patients with IMA, thoughtful surveillance after curative treatment and timely management upon recurrence could help improve treatment outcomes. Therefore, understanding the dynamics of recurrence and death according to specific prognostic factors is essential for overcoming the dismal prognosis of IMA. Traditional survival estimates are given by survival from the time of diagnosis in most reports, representing cumulative survival. As a result, cumulative survival estimates calculated at the time of initial diagnosis have limited utility for follow-up care, since they provide only a static view of risk without postoperative follow-up information and do not reflect changes in prognosis over time. The hazard rate curve is more relevant to follow-up care because it reflects the change of survival likelihood with increasing duration of follow-up after curative operation for the initial cancer. To the best of our knowledge, this is the first series to investigate the pattern and dynamics of recurrence using hazard rates in patients with solitary IMA who received surgical therapy.

A notable result of our study is that consolidative CT morphology was a significant, poor prognostic factor for DFS and OS. With improvements in CT image quality, correlations between CT and pathologic findings are more often noted, and imaging is recognized as a valuable tool for providing prognostic information [5, 7, 28]. Lee et al. found alveolar spaces filled with mucin and mononuclear cells and alveolar walls lined by mucin-containing tumor cells account for the solid or part-solid nodular features of IMAs [14]. Also, they reported lobulated and spiculated margins were associated with STAS, with probable characteristic imaging features focused on IMA. Lobulated and spiculated margins reflect the interaction between tumor growth and normal lung parenchyma [12]. Also, other several previous studies demonstrated that pneumonic type IMA reflected alveoli filled with abundant mucin and tumor cells with mucin spread aerogenously throughout the alveoli and is correlated with poorer prognosis compared with the nodular type [8, 10–13]. Cha et al. hypothesized that tumor cells travel in the background of abundant alveolar mucin and become situated in the alveolar walls away from the primary lesion [29]. Accordingly, as abundant alveolar mucin and tumor cells with spread aerogenously throughout the alveoli might be seen as consolidative lung parenchymal opacity on CT, we hypothesized that pneumonic type IMA itself may reflect higher potential for microscopic tumor spread. Moreover, the diagnosis of pneumonic type recurrent IMA is often delayed because of the intrinsic difficulty in distinguishing infectious pneumonia and pneumonic type IMA [8–11], and this difficulty might affect the results of prognostic factor analysis. Considering the CT morphology is an important factor in prognosis, it should be very reproducible across observers and across institutions. Thus, we calculated the interobserver agreement between the two readers for CT morphology and it was substantial (kappa value of 0.78). Since there is no portion of this study that looks at the reproducibility of the classifications of CT morphology, this favorable results of reproducibility acknowledge that radiologic factors might be useful for prognostication of IMA.

In recent years, limited resection techniques such as wedge resection and segmentectomy with less hilar/mediastinal LN dissection have become important treatments for patients with stage IA non-small cell lung cancer (NSCLC) in accordance with developing video-assisted thoracic surgery [30, 31]. However, recent studies have suggested that in patients with STAS, limited resection was associated with a significantly higher risk of recurrence than lobectomy [32, 33]. In line with the results of previous reports, in this study we found that STAS was a poor independent prognostic predictor for OS rate in IMA patients. STAS showed a negative impact on prognosis based on the 2015 World Health Organization (WHO) Classification for lung adenocarcinoma in two large cohort studies [15, 16, 34]. In addition, according to a recent meta-analysis, the presence of STAS is a poor prognostic factor for patients with NSCLC [35]. Therefore, we agree that the presence of STAS is an independent poor prognostic factor. A higher incidence of intrapulmonary metastasis without extrapulmonary metastasis has been reported in patients with IMA [36]. Likewise, we also observed that the majority of patients who had distant metastasis showed only intrapulmonary metastasis. The characteristics of intraalveolar tumor cells, which indicate STAS, with detached primary focus raises the possibility of a pathogenic association with intrapulmonary metastasis. Although there was no relationship between surgical procedure and prognosis in our study, decisions regarding whether to proceed with limited resection should be carefully taken for patients with IMA, considering the higher incidence (50%–72.3%) of STAS than in other types of adenocarcinomas [12, 18, 19]. However, our suggestion should be confirmed by further studies due to its conflicting results.

In this study, hazard rate curves for recurrence and death according to independent prognostic factors revealed that the peaks of the hazard rate curves appeared in the earliest phase during the follow-up period and declined rapidly. Lou et al. [37] found that approximately one-third of recurrences occurred within two years after resection in patients with early-stage lung cancer, and the high risk of recurrence persisted for up to four years after resection. Several other studies of the dynamics of recurrence after treatment for early or stage IIIA NSCLC demonstrated that the risk of recurrence is not constant over time [23, 38–40]. Rather, multiple peaks are observed in hazard rate analysis, and these patterns vary according to clinical or pathological factors such as sex, pathological nodal stage, and pattern of recurrence. In the present study, we obtained results agreeing broadly with conventional ideas, but not entirely because recurrence occurred most frequently during the earliest phase, that is, within three months after resection (the first visit), and this is a novel finding for IMA. But, in previous studies, the first peak of recurrence usually occurred after approximately 9–12 months for similar stage of NSCLC [23, 38–40]. This finding may justify the emphasis on intensive surveillance during the first follow-up visit period. Although there is not certain data supporting our hazard rate results, we assumed that the higher incidence of STAS in IMA [41] may contribute the earliest recurrence. Further studies for validating our results should be needed. We also observed that the risk of recurrence or death displayed steeper decline, but persisted for up to 4 years after surgery as in previous studies for NSCLC. This finding suggests an earlier and more clustered presentation of recurrence or death could be observed in patients with IMA treated with surgical resection. Even though our results did not show far distinguishable points on hazard rate analysis compared with previous studies, we think these results are meaningful because distinct tumor characteristics of IMA make predicting its recurrence dynamics difficult and it is hard to expect identical hazard rate curve to other major subtypes of adenocarcinomas. Thus, we believe it helps in terms of building baseline research data for the next relevant study. Additionally, we observed recurrence subtype-specific (oligometastases vs. polymetastases) survival dynamics. The hazard rate curve demonstrated that the peak of recurrence differed among different subtypes of metastases, with polymetastases having higher hazard rates for death than oligometastases and death peaking at 40 months after surgery. During the past decade, the use of surgery or focally ablative therapies such as stereotactic ablative radiation therapy (SABR, also known as stereotactic body radiation therapy) for oligometastases has increased in several types of cancers, including lung cancer, for curative-intent treatment [42–44]. Our results confirm the general idea that patients with oligometastases who underwent ablative treatments can achieve “better-than-expected” long-term survival in IMA.

Our study had several limitations. This was a retrospective study performed at a single institution. External validation is required to consolidate the results of our study in a larger series of patients. However, to overcome this limitation, we aimed to include as many IMA patients as possible and reviewed all formalin-fixed slides of resected IMAs at our institution collected during a period longer than 13 years. Second, the differences in follow-up protocols, which vary depending on the physician, could skew the survival data. Additionally, surveillance scans were performed at predefined intervals. Recurrence may develop between clinical visits. Third, although we tried to include all available consecutive IMA patients, the imbalanced distributions of various clinicopathological and radiological variables may influence the generalizability of our results. Additional larger and more balanced validation studies are warranted in the future. Lastly, approximately ¼ of the patients received adjuvant or neoadjuvant therapy while ¾ did not. This creates an inhomogeneous patient population which is a limitation of this study. However, IMA is a rare tumor, so it is difficult to gather such a large population as our study. Moreover, IMAs which can present consolidative morphology tend to be larger than nonmucious adenocarcinomas in extent, and that's why many patients had received adjuvant management. In our study, we used whether patients received adjuvant/ neoadjuvant therapy or not as an input variable in univariate and multivariate analysis for DFS and OS, and found that there was no significant difference in prognosis according to the adjuvant/neoadjuvant therapy. Thus, significant independent prognostic factors (consolidative morphology on CT, higher T and N stage, smoking, and STAS) were all adjusted to the adjuvant/neoadjuvant therapy.

In conclusion, IMA patients with consolidative CT morphology, higher T stage, and higher N stage were significantly more prone to recurrence, while history of smoking, presence of STAS, consolidative CT morphology, higher T stage, and higher N stage were significantly associated with worse OS. The dynamics of recurrence and death after surgical treatment showed higher hazard rates and peaks that appeared during the early phase of follow-up according to significant clinicopathologic and radiologic prognostic factors. Our findings provide information relevant to the selection of patients at higher risk of recurrence and death as well as the timing of surveillance studies.

## Supplementary Information

**Additional file 1**. **Figure S1**. Receiver operating characteristic (ROC) analysis for the prediction of disease-free survival (DFS) and overall survival (OS). **Figure S2**. Receiver operating characteristic (ROC) with five‐fold cross‐validation of the sensitivity and specificity for the prediction of disease-free survival (DFS) and overall survival (OS) at several time points. **Figure S3**. The calibration plot of the prediction models for (A) disease-free survival (DFS) and (B) overall survival (OS) at 36 and 60 months. **Figure S4**. Comparison of Kaplan–Meier curves of DFS according to CT morphology (A), T stage (B) and N stage (C) in patients with invasive mucinous adenocarcinoma. **Figure S5**. Comparison of Kaplan–Meier curves of OS according to smoking (A), STAS (B), consolidative CT morphology (C), higher T stage (D), higher N stage (E), and recurrence subtype (F) in patients with invasive mucinous adenocarcinoma. **Figure S6**. Comparison of recurrence hazard rate according to STAS in patients with invasive mucinous adenocarcinoma. **Table S1**. Detailed follow-up information with demographic and tumor characteristics in the eight patient who underwent sublobar resection.

## Data Availability

The datasets used and/or analyzed during the current study are available from the corresponding author on reasonable request.

## Abbreviations

AUC: Area under the curve
CCRT: Concurrent chemoradiotherapy
CT: Computed tomography
DFS: Disease-free survival
EGFR: Epidermal growth factor receptor
GGO: Ground-glass opacity
HR: Hazard ratio
IMA: Invasive mucinous adenocarcinoma
IQR: Interquartile range
LN: Lymph node
NSCLC: Non-small cell lung cancer
OS: Overall survival
ROC: Receiver operating characteristic
STAS: Spread through air spaces
SABR: Stereotactic ablative radiation therapy
WHO: World Health Organization
