# Supplementary material for: Recurrence dynamics after curative surgery in patients with invasive mucinous adenocarcinoma of the lung
Source: Insights Imaging. 2022 Apr 5;13:64. doi: 10.1186/s13244-022-01208-5 (PMC8982735; doi:10.1186/s13244-022-01208-5)
Supplement: Supplementary file 1 — Additional file 1. Figure S1. Receiver operating characteristic (ROC) analysis for the prediction of disease-free survival (DFS) and overall survival (OS). Figure S2. Receiver operating characteristic (ROC) with five‐fold cross‐validation of the sensitivity and specificity for the prediction of disease-free survival (DFS) and overall survival (OS) at several time points. Figure S3. The calibration plot of the prediction models for (A) disease-free survival (DFS) and (B) overall survival (OS) at 36 and 60 months. Figure S4. Comparison of Kaplan–Meier curves of DFS according to CT morphology (A), T stage (B) and N stage (C) in patients with invasive mucinous adenocarcinoma. Figure S5. Comparison of Kaplan–Meier curves of OS according to smoking (A), STAS (B), consolidative CT morphology (C), higher T stage (D), higher N stage (E), and recurrence subtype (F) in patients with invasive mucinous adenocarcinoma. Figure S6. Comparison of recurrence hazard rate according to STAS in patients with invasive mucinous adenocarcinoma. Table S1. Detailed follow-up information with demographic and tumor characteristics in the eight patient who underwent sublobar resection. [file 13244_2022_1208_MOESM1_ESM.docx]

Figure S1. Receiver operating characteristic (ROC) analysis for the prediction of disease-free survival (DFS) and overall survival (OS).


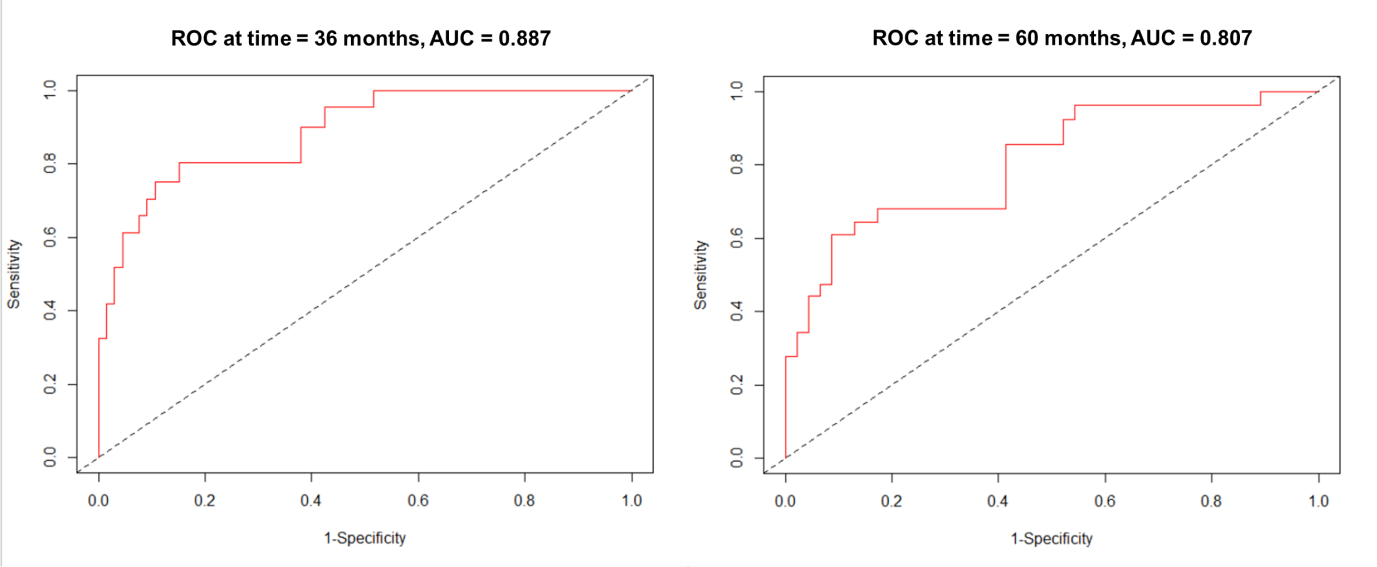


(A)


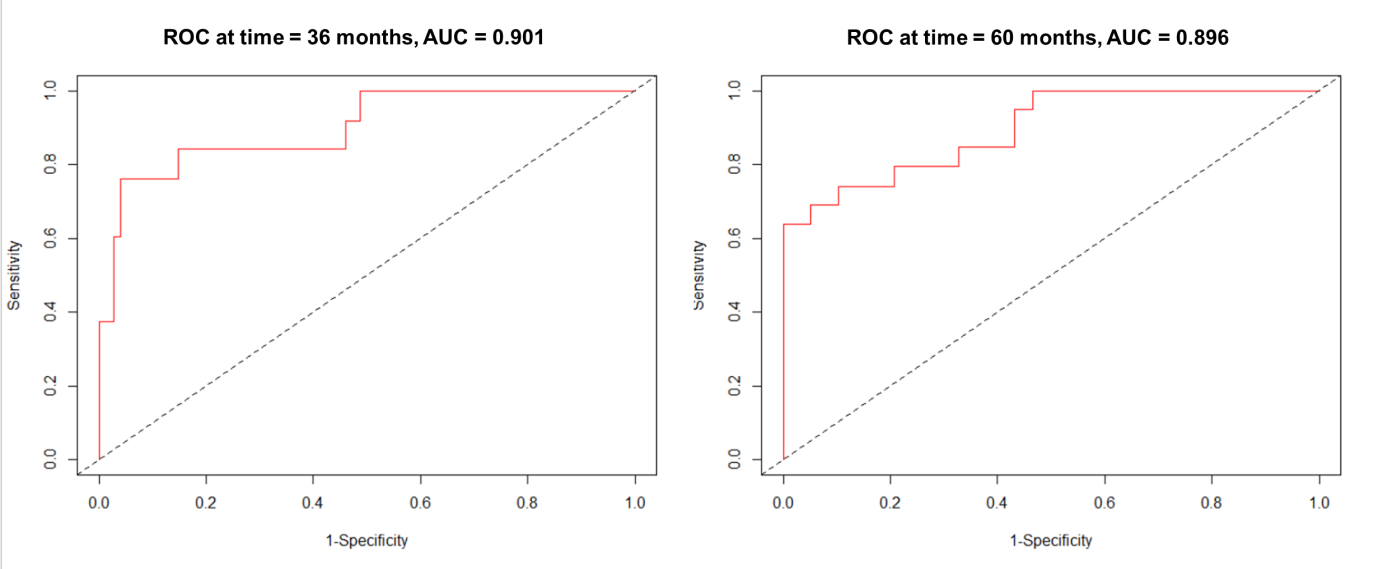


(B)

(A) The predictive model for prediction of DFS showed performance with area under the receiver operating characteristic curve (AUC) of 0.887 at 36 months and 0.807 at 60 months. (B) The predictive model for prediction of OS showed performance with AUC of 0.9 at 36 months and 0.896 at 60 months.


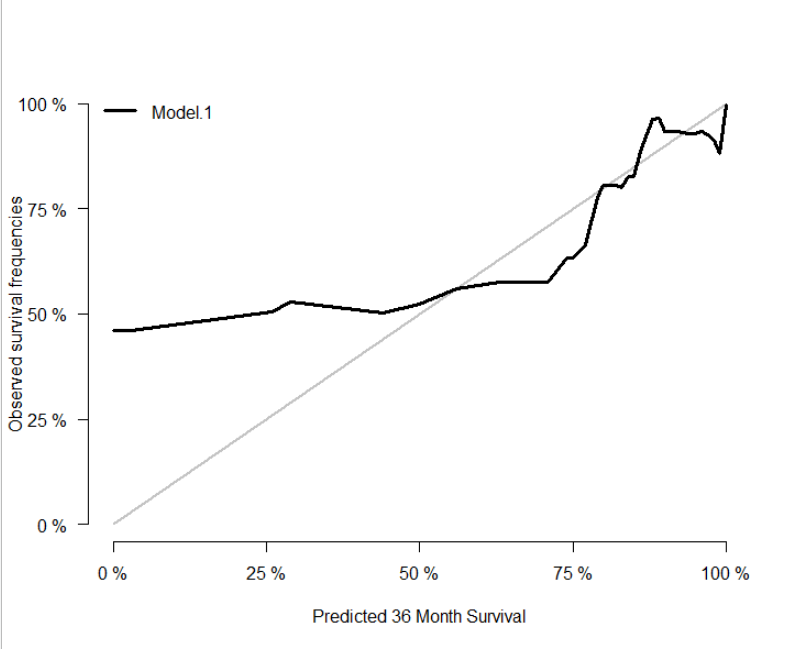

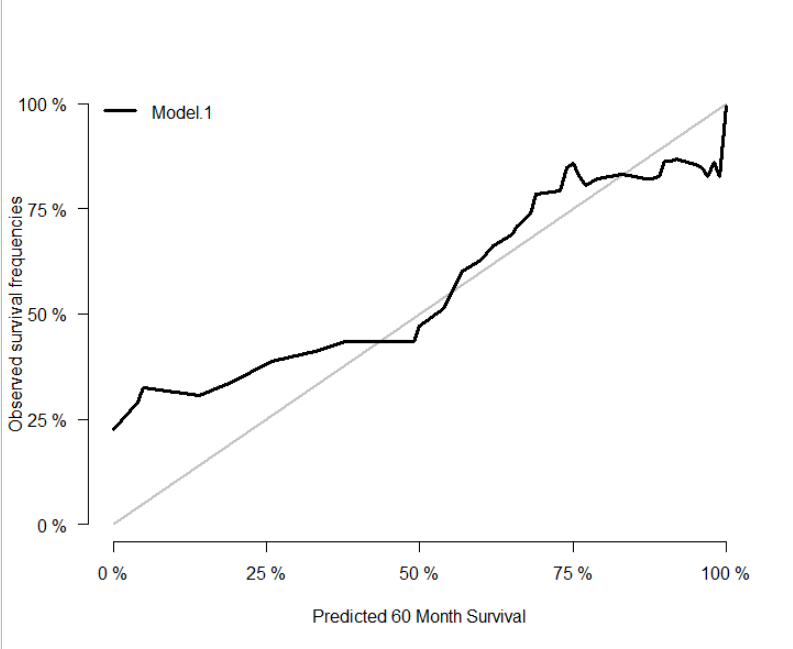
Figure S2. Receiver operating characteristic (ROC) with five‐fold cross‐validation of the sensitivity and specificity for the prediction of disease-free survival (DFS) and overall survival (OS) at several time points.


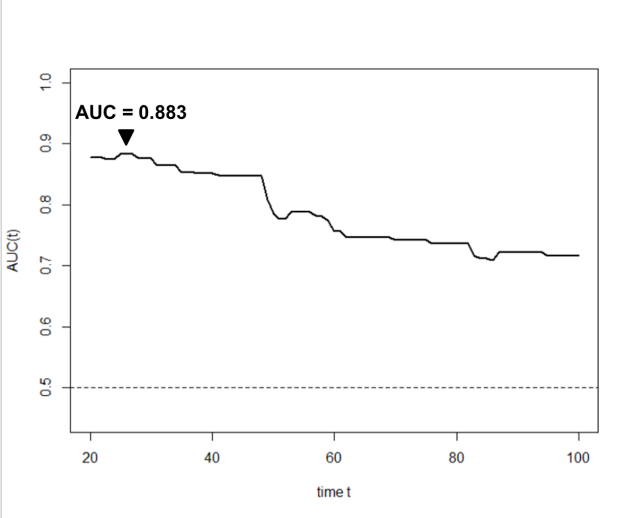

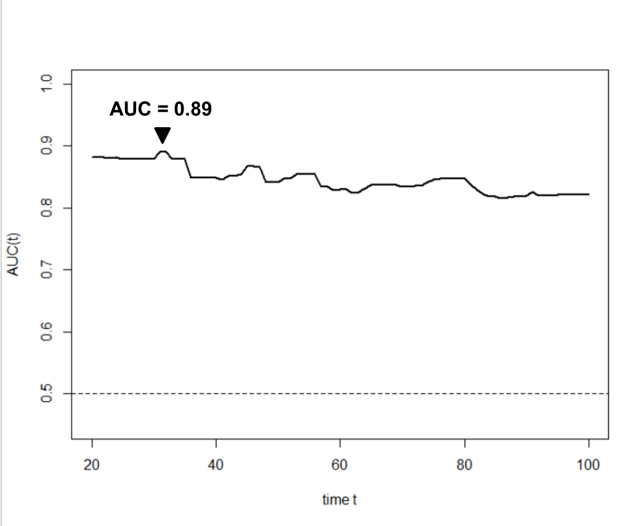


1. (B)

(A) Area under the receiver operating characteristic curve (AUC) of 0.883 was highest at 25, 26 and 27 months for DFS, and (B) AUC of 0.89 was highest at 31 and 32 months for OS.

Figure S3. The calibration plot of the prediction models for (A) disease-free survival (DFS) and (B) overall survival (OS) at 36 and 60 months.


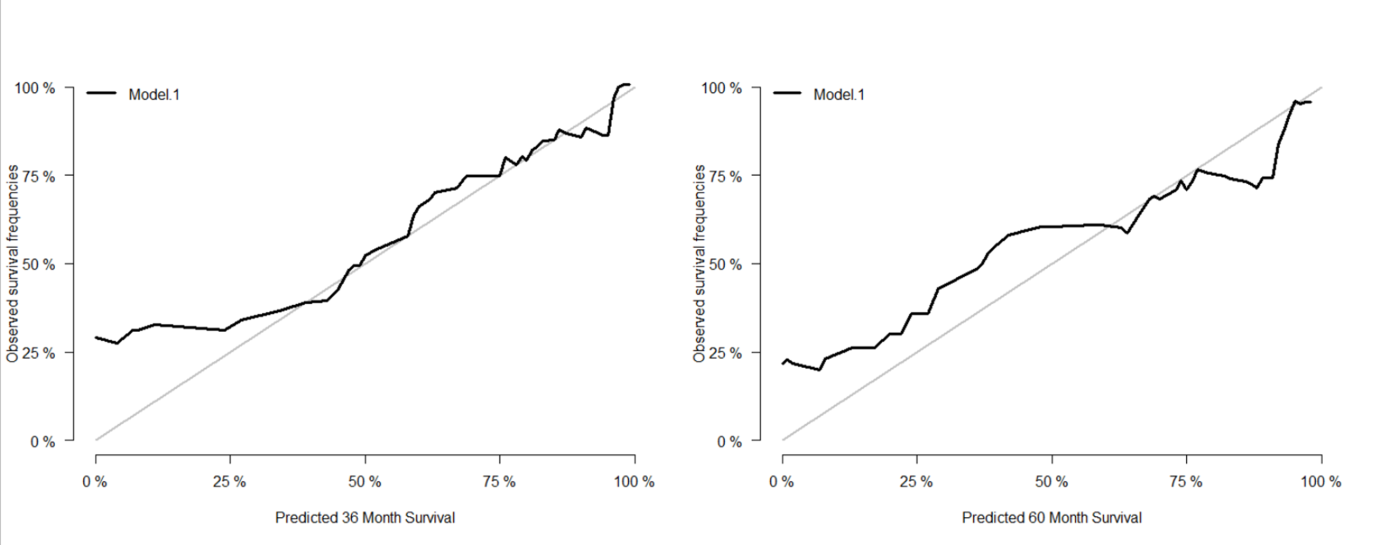


(A)


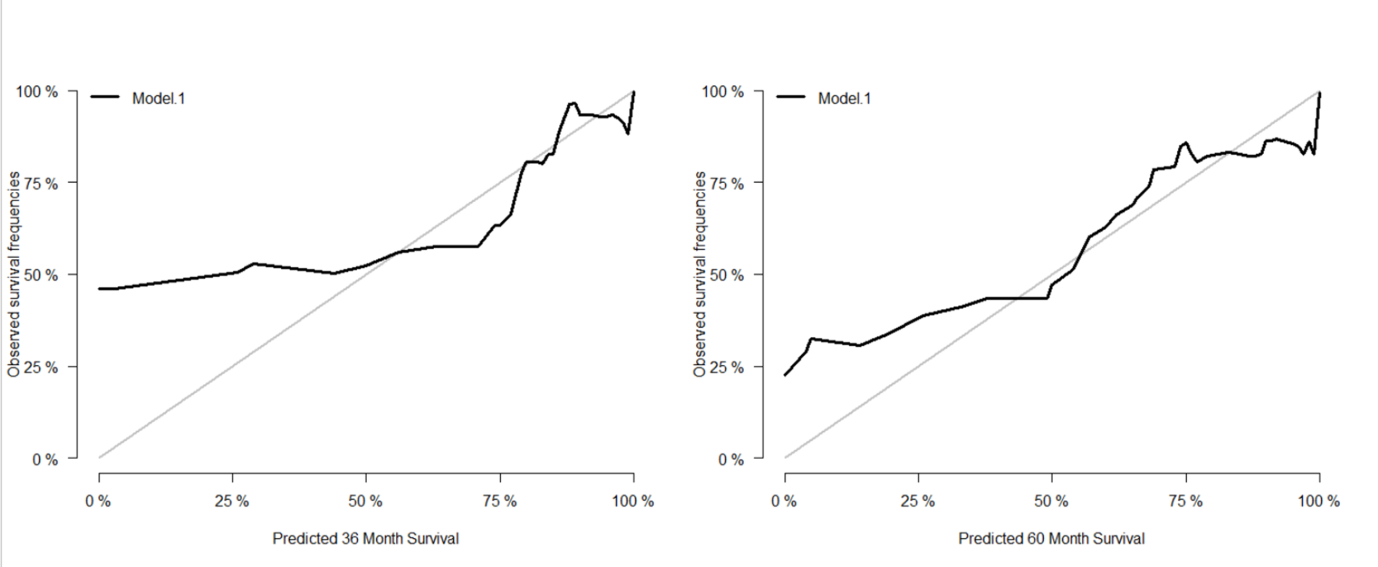


(B)

Figure S4. Comparison of Kaplan–Meier curves of DFS according to CT morphology (A), T stage (B) and N stage (C) in patients with invasive mucinous adenocarcinoma.


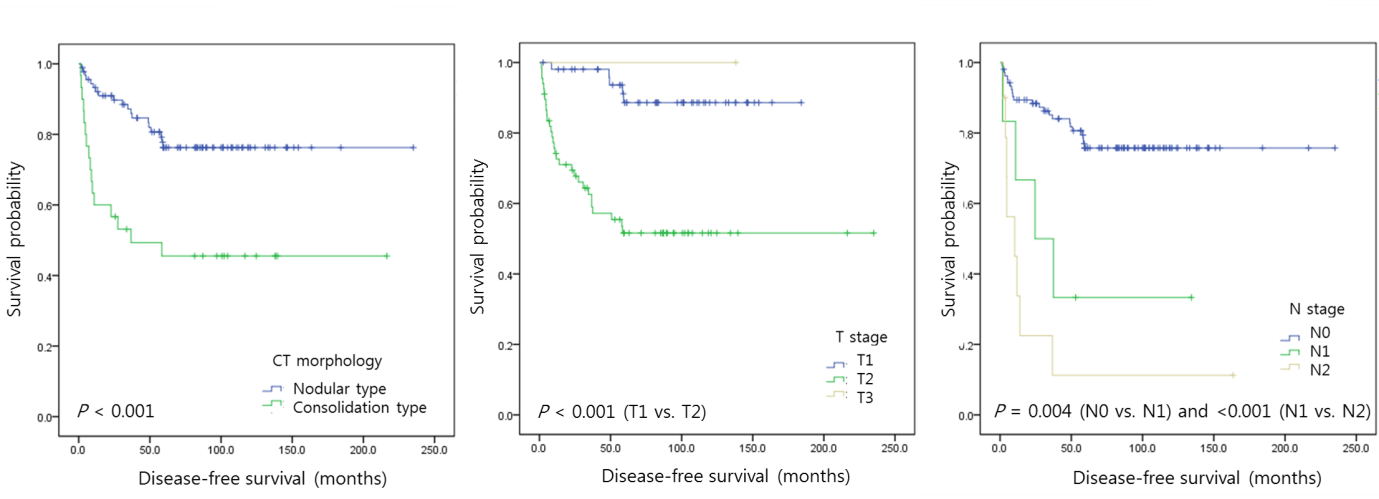
(A) (B) (C)

Figure S5. Comparison of Kaplan–Meier curves of OS according to smoking (A), STAS (B), consolidative CT morphology (C), higher T stage (D), higher N stage (E), and recurrence subtype (F) in patients with invasive mucinous adenocarcinoma.


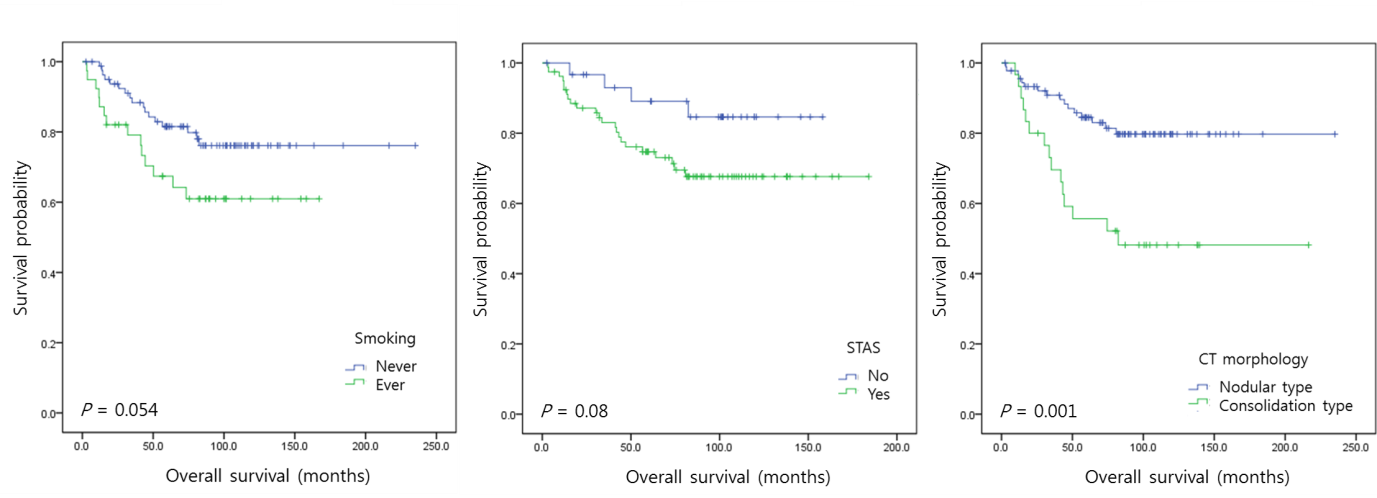
(A) (B) (C)


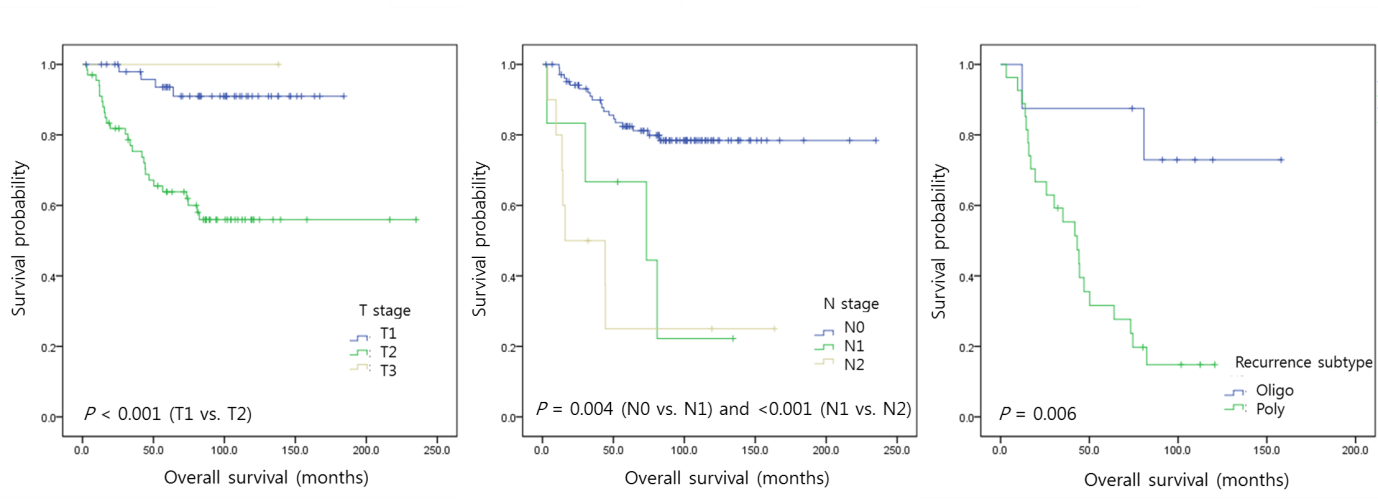
(D) (E) (F)

Figure S6. Comparison of recurrence hazard rate according to STAS in patients with invasive

mucinous adenocarcinoma.


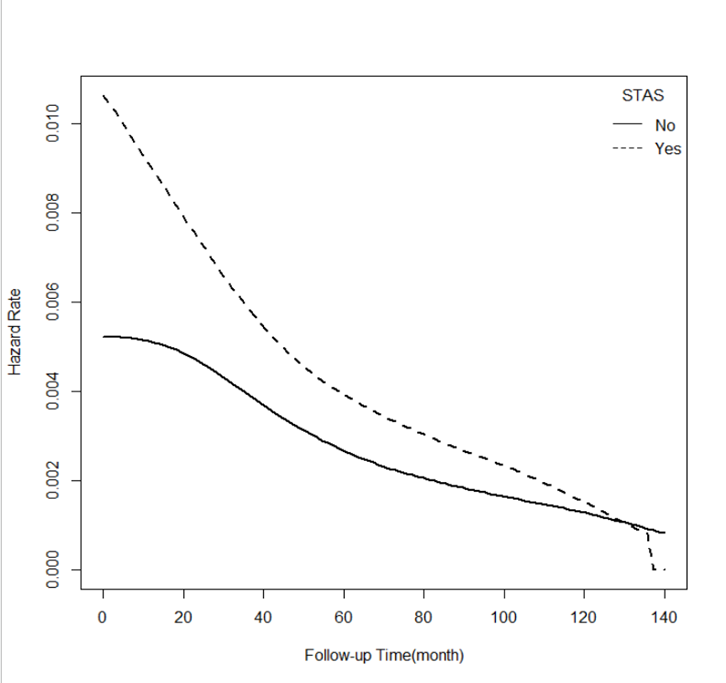


Table S1. Detailed follow-up information with demographic and tumor characteristics in the eight patient who underwent sublobar resection

| Patient  No. | Age | Sex | History of smoking | T stage | N stage | Tumor differentiation | STAS | CT morphology | OS  event | DFS event | Follow-up  period(months) |
| --- | --- | --- | --- | --- | --- | --- | --- | --- | --- | --- | --- |
| 1 | 64 | Male | No | T2 | N0 | Well | Yes | Nodular | No | No | 86.4 |
| 2 | 54 | Female | No | T1 | N0 | Well | No | Nodular | No | No | 151.1 |
| 3 | 65 | Male | No | T1 | N0 | Well | Yes | Nodular | No | No | 108.2 |
| 4 | 66 | Female | No | T1 | N0 | Well | No | Nodular | No | No | 40.8 |
| 5 | 58 | Male | Yes | T2 | N0 | Well | No | Consolidative | No | No | 100.6 |
| 6 | 60 | Male | Yes | T1 | N0 | Well | No | Nodular | No | No | 101.6 |
| 7 | 66 | Male | Yes | T1 | N0 | Well | Yes | Nodular | No | No | 75.5 |
| 8 | 56 | Female | No | T1 | N0 | Well | No | Nodular | No | No | 61.1 |

STAS, spread through air spaces; OS, overall survival; DFS, disease-free survival
